# Dose-dependent biological toxicity of green synthesized silver nanoparticles in rat’s brain

**DOI:** 10.1038/s41598-022-27171-1

**Published:** 2022-12-31

**Authors:** Mai Tareq, Yasser A. Khadrawy, Monira M. Rageh, Haitham S. Mohammed

**Affiliations:** 1 Biophysics Department, Faculty of Science, Cairo University, Giza, Egypt; 2 Medical Physiology Department, Medical Research and Clinical Studies Institute, National Research Centre, El-Bohouth St, Giza, Egypt

**Keywords:** Biochemistry, Biophysics

## Abstract

Metal nanoparticles, in general, and silver nanoparticles (AgNPs), in particular, have been the focus of numerous studies over the last two decades. Recently, the green synthesis of metal nanoparticles has been favored over chemical synthesis due to its low toxicity and easy preparation. The present study aims to investigate the dose-dependent toxicity of green synthesized AgNPs on rats’ brains. Thirty-four Wistar male rats were divided into four groups. The first, second, and third groups were administered for 14 days with three different doses (0.5, 5, and 10 mg/kg) of AgNPs, respectively. The fourth group, which served as a control group, was given normal saline for the same period. The toxicity of the green synthesized AgNPs on the cortical and hippocampal levels of the oxidative stress markers (MDA, NO, and GSH) as well as the activity of acetylcholinesterase (AchE) and the monoamine neurotransmitters (DA, NE, and 5H-T) were investigated. AgNPs showed minimal oxidative stress in the cortex and hippocampus for the administered doses. However, AgNPs showed an inhibitory effect on AchE activity in a dose-dependent manner and a decrease in the 5H-T and NE levels. The green synthesized AgNPs showed an ultrastructural change in the cellular membranes of the neurons. The green synthesis of AgNPs has reduced their cytotoxic oxidative effects due to their capping with biologically compatible and boosting molecules such as flavonoids. However, another neurotoxicity was observed in a dose-dependent manner.

## Introduction

Metal nanoparticles and specifically silver nanoparticles (AgNPs) have been the center of numerous research for the past decades. This interest rises from their unique and remarkable physicochemical properties, which are attributed mainly to their large surface area to volume ratio and its consequent properties such as surface charge and agglomeration. All these factors contribute greatly to their effects in the different fields whether environmentally, industrially, or medically^1^. AgNPs have been proven to have unique antimicrobial toxicity along with other advantageous toxicity like anticancer and catalytic toxicity^2–6^, consequently, they are widely used in fields of water purification, products manufacturing that have antibacterial features, in coatings of medical devices, and as antiseptics^7^.

With the extensive use of AgNPs, the exposure rate of humans and other organisms in the environment to these nanoparticles is elevated massively. Consequently, the concerns about the health hazards of these nanoparticles also rise, especially when they are reported to accumulate and persist for a long time in biological tissues like the brain and testes^8^. The lack of the full profile of the biological toxicity of AgNPs on humans and other living organisms necessitates the investigation of these toxicities to gain the benefits and avoid the hazards of using these nanoparticles.

The toxicity of AgNPs on biological systems could be attributed to multiple parameters, such as their size, shape, coating, dose, agglomeration, and synthesis method. Therefore, the difference in the results obtained from different investigations may be due to the difference in these parameters. Different nanoparticle synthesis methods, chemically, physically, or biologically, have proved to affect the biological systems in different ways. The biological methods of AgNPs synthesis have displayed the highest benefit and the least toxic effects when used with appropriate dosing^6^. It was reported that AgNPs cause toxicity by inducing oxidative stress and apoptosis^9–11^. Other studies suggested that AgNPs cause genotoxicity^12^. These toxicities have been documented in in-vitro and in-vivo studies^13,14^.

The role of monoamine neurotransmitters in modulating brain function is well known. However, the toxicity of metal nanoparticles on the levels of these neurotransmitters in different brain regions are rarely investigated. Whether the toxicity on these monoamines are due to direct interaction with the metal nanoparticles^15^, or indirectly through other mechanisms is still partially understood. Due to the major role played by neurotransmitters in brain function and their consequences on the whole body, studying the toxicity of the widely used nanoparticles such as AgNPs on neurotransmitter systems is of paramount importance.

Several studies investigated the toxicities or effects of biosynthesized AgNPs from *Psidium guajava*, however, they were mainly focusing in investigated their effects on microorganisms as bacteria or fungi^16–21^. Furthermore, other studies reported findings related to the impact of AgNPs on brain tissues, yet were mostly not biosynthesized^22–27^. Therefore, in the present study we intended to investigate the biologically synthesized sliver nanoparticles on brain tissues.

The present work aimed to evaluate the in-vivo toxicity of *Psidium guajava* leaf extract (PGLE) synthesized-AgNPs on the brain of the experimental animals. *Psidium guajava* leaves were chosen in the present study, as they are known for their medicinal properties due to their rich content of flavonoids and for being available with an easy access. After characterization and size optimization of AgNPs, three different doses (low, medium, and high) were selected to investigate the dose-dependent toxicity of the synthesized nanoparticles on two different brain regions; cortex and hippocampus. To this end, oxidative stress markers, monoamine neurotransmitters levels, acetylcholinesterase activity, and tissue’s structural aspects were investigated.

## Materials and method

### Materials and chemicals

Fresh matured leaves of *Psidium guajava* were collected from a local botanical garden in Cairo University. University permits the researchers to collect plant leaves according to the international guidelines and legislation. Only 7 to 9 leaves were used during each preparation of the nanoparticles. To maintain the freshness of the leaves they were stored in a clear plastic bag in the lab refrigerator at 4 °C. Silver nitrate (AgNO_3_, 99.8–100% pure), phosphate buffer, and acetylthiocholine iodide were purchased from Sigma-Aldrich (Merk, St. Louis, MO, USA). Thiobarbituric acid (TBA), Trichloroacetic acid, 5,5′dithiobis (2-nitrobenzoic acid), Sulfanilamide, and N-(1-naphthyl)ethylenediamine were purchased from Biodiagnostic Co., Giza, Egypt.

### Preparation of plant extract

Psidium guajava leaf extract (PGLE) was prepared as described by Bose and Chatterjee^20^ with slight modifications including doubling the amount of ingredients. The leaves were thoroughly washed under tap water and then soaked and washed with distilled water to remove any adsorbed dust and other particles. The leaves were then left to dry at room temperature on filter papers. 10 g of leaves were weighed and put in a small kitchen chopper with 120 mL of distilled water. Leaves were crushed by the chopper for almost 30 s until a green solution of very fine pieces of leaves was produced. The produced mixture was then filtered using Whatman No.1 filter paper. The yellowish filtrate was then poured into test tubes and stored in the freezer as a stock solution for further use during the experiment.

### Synthesis of silver nanoparticles

AgNPs were synthesized according to Bose and Chatterjee^20^ with some modifications as follows: 20 ml of a 5 mM AgNO_3_ solution was poured into a 50 ml Erlenmeyer flask and 0.2 ml of PGLE was added to the 20 ml of AgNO_3_ solution. The mixture was heated to 90 °C and stirred at 200 rpm for 20 min on a hotplate magnetic stirrer (MSH-20D, Daihan Scientific, Wonju, South Korea). The color of the stirred solution changed from colorless to dark orange or light brownish color, which confirmed the synthesis of the AgNPs and indicated the reduction of the AgNO_3_ solution by the PGLE to produce the AgNPs. The color of the produced silver nanoparticle aqueous solution was darkened with time, producing darker reddish-brown color indicating a further reduction of AgNO_3_ by the PGLE solution till its completion.

### Size optimization of biosynthesized AgNPs

Optimization of AgNPs was important to control their size which would affect their BBB entry and concentration in the administered doses. Optimization of AgNPs was accomplished via the change in the following parameters: molarity, pH, temperature, and volume of PGLE during the synthesis process. Different molarities of AgNO_3_ solution were investigated to optimize the particle size, these were: 1 mM, 3 mM, 5 mM, 10 mM, 13 mM, and 15 mM. Different pH values of the AgNO_3_ solution were investigated to optimize the particle size, these were pH: 6, 7, 9, 11, and 12. The pH was controlled by 0.1 N NaOH. The particle size was also optimized by synthesizing the AgNPs at different temperatures: 30 °C, 50 °C, 70 °C, and 90 °C while keeping other parameters constant. The PGLE volume was also changed through the synthesis trials to optimize the particle size. The extract volumes investigated were: 0.2, 0.4, 0.6, 0.8 and 1 mL that were added to the 20 mL of AgNO_3_ solution. The different particle size optimization parameters are summarized in Table 1.

**Table 1:** AgNPs size optimization determined by DLS at different AgNO_3_ molarities, pH, temperatures, and volumes of the added PGLE.

Average size by DLS	Polydispersity index (PDI)	Molarity (nM)	pH	Temperature (^o^C)	PGLE Volume (mL)
552.2	0.816	15	7	90	0.2
141.6	0.373	1	12	90	0.2
138.1	0.299	1	7	70	0.2
134	0.489	1	7	50	0.2
128	0.598	1	7	30	0.2
111.6	0.302	13	6	90	1
110.8	0.432	1	9	90	0.2
71.66	0.219	13	7	90	0.8
71.21	0.195	1	7	90	0.2
70.88	0.427^a^	5^a^	7^a^	90^a^	0.2^a^
65.43	0.161	13	7	90	0.2
64.44	0.218	13	7	90	0.4
61.19	0.238	13	7	90	1
57.91	0.207	3	7	90	0.2
37.57	0.467	1	11	90	0.2

^a^Chosen parameters for the synthesis of the administered AgNPs doses in rats as optimum conditions due to the production of the optimum AgNPs size, concentration, and stability.

### Characterization of silver nanoparticles

The size, shape, and surface charge of the AgNPs along with other factors play an important role in their toxicity on the biological system; therefore, the characterization of the synthesized nanoparticles is important to determine their shape, size, and surface area properties, and stability. The characterization of the synthesized and optimized AgNPs was done through the following techniques: UV–Vis spectrophotometry, Fourier transform infrared spectroscopy (FTIR), Transmission electron microscopy (TEM), and Dynamic light scattering (DLS).

### UV–Vis spectroscopy

AgNPs have a unique absorption peak known as the surface plasmon resonance (SPR) peak which usually occurs, for the PGLE biosynthesized particles, in the range of 419–460 nm^18–20,28^. This peak is characteristic of the silver nanoparticles which could be used as a confirmation for the synthesis of silver nanoparticles; hence the absorption peak of the PGLE was also measured in the same range for further confirmation. The UV–Vis spectroscopy was carried out in the range of wavelengths of 350–650 nm on a UV–Vis Spectroscopy (Alpha-1502, Shanghai Lab-spectrum, instruments Co, Ltd).

### Fourier transform infrared spectroscopy

The structures and compositions of the dried powder of the AgNPs, AgNO_3,_ and PGLE were measured and analyzed using the FTIR technique in the range of 400–4000 cm^−1^ at a resolution of 4 cm^−1^ using an FTIR Spectrometer (FT/IR-4100 type A, JASCO, Japan).

### Transmission electron microscopy

The size and morphology of the particles were determined by transmission electron microscope imaging using JEOL, JEM-1230, high performance, high contrast, 40–120 kV, transmission electron microscope (TEM). The particles were placed on a carbon grid to be examined and captured. The size distribution of the captured particles was done by the IMAGEJ SOFTWARE.

### Dynamic light scattering

The hydrodynamic size, size distribution, and zeta potential of the produced nanoparticles were measured by the dynamic light scattering device (Malvern Zetasizer, nano-Zs 90, Malvern Instruments Ltd., UK).

### Experimental animals

Thirty-four adults male Wistar rats were used in the present study. Their average weight was 152 ± 20 g. The animals were purchased from the National Institute of Cancer, Cairo, Egypt. On arrival, they were left for 1 week to acclimatize before the experimental procedures. They were maintained in standard environmental conditions of humidity, temperature, and 12 h day and night cycles. The animals had access to food and water ad libitum.

### Experimental design

**Figure 1:**
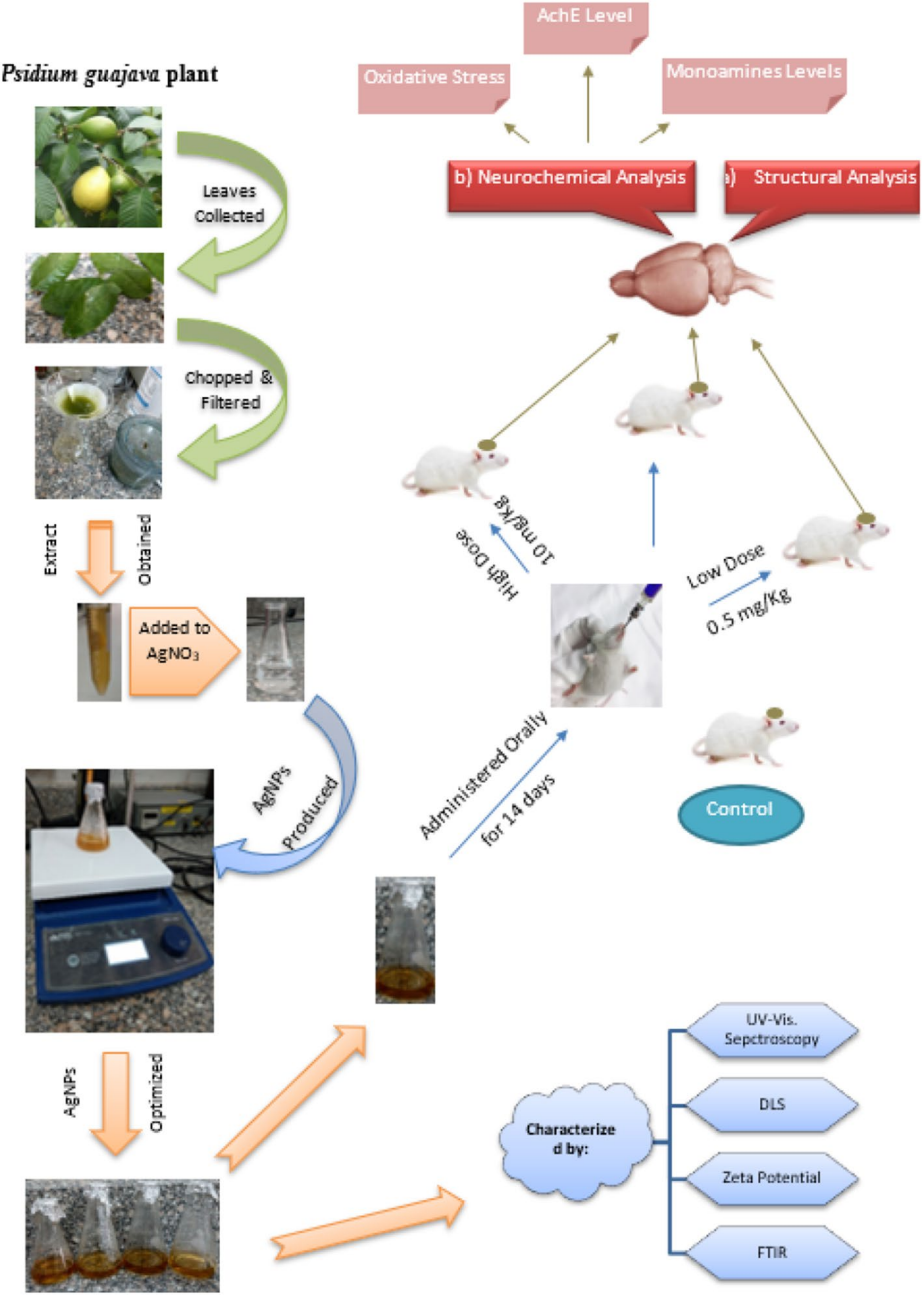
Graphical representation of the experimental procedures and study design and evaluation parameters.

The animals were divided into four groups, control (n = 10) received orally 0.9% saline solution, the second, third, and fourth groups (n = 8/group) received orally AgNPs at 0.5, 5, 10 mg/kg, respectively. All rats in different groups were administered daily for 14 days (Fig. 1). At the end of the experiment, the animals were sacrificed by sudden decapitation on the 14th day, one hour after the last administration. The brain of each rat was dissected and divided into two halves. The right half of the brain of 3 animals, from different groups, was separated for the ultrastructural transmission electron microscope (TEM) imaging. In the rest of the animals, the cortex and hippocampus brain regions were dissected. Each brain region was weighed and kept frozen at − 30 °C until the biochemical analyses.

### Ultrastructural examination of brain tissues by electron microscope

The ultrastructural examination was carried out to detect the presence of AgNPs in brain tissues by TEM. The samples were preserved in ice-cold glutaraldehyde solution as a first fixation step for examination. The tissues were then rinsed and fixed again with osmium tetroxide for better contrast of the image. Then they were dehydrated by different concentrations of ethanol and eventually embedded in epoxy resin to make them firm enough to handle the pressure of the cutting process. Ultra-thin sections, at approximately 75–90 μm in thickness, were obtained from the prepared resin by a Leica EM UC6 ultramicrotome (Leica Microsystems Co.) and examined by TEM, JOEL (JEM-1400 TEM) at direct magnification 2500× to 25,000× at HV = 80.0 kV with a scale bar of 500 nm–2 mm.

### Neurochemical analysis

Neurochemical analyses were carried out on the hippocampus and cortex areas of the rat’s brain. Each brain area was homogenized in ice-cold tris hydrochloric acid (tris HCL) buffer of pH 7.4. The homogenate was then centrifuged for 30 min at 4 °C at 5000 rpm in a high-speed cooling centrifuge (Type 3k-30, Sigma, Germany). Then the following parameters were measured.

#### Oxidative stress parameters

##### Lipid peroxidation

Lipid peroxidation was measured according to the method of Ruiz-Larrea et al.^29^. This method depends on measuring malondialdehyde (MDA) as an indicator of lipid peroxidation. A 200 μL of the tissue homogenate was added to a 1000 μL of the thiobarbituric acid (TBA) (chromogen) and heated in a boiling water bath for 30 min, then left to cool. The absorbance of the resultant solution was measured at 534 nm.

##### Reduced glutathione

Reduced glutathione (GSH) was measured on the homogenates of the hippocampus and cortex using the method of Beutler et al.^30^. A 250 μL of the tissue homogenate was added to a 250 μL of trichloroacetic acid, mixed well, allowed to stand for 5 min, and centrifuged at 3000 rpm for 15 min. 250 μL of the supernatant was then added to a 500 μL buffer solution with 250 μL of 5,5′dithiobis (2-nitrobenzoic acid). The absorbance of the resultant solution was measured at 405 nm.

##### Nitric oxide

The method used to measure nitric oxide (NO) uses Griess reagent based on the method of Montogomery and Dymock^31^. A 100 μL of tissue homogenate was added to a 1000 μL of sulphanilamide and left to stand for 5 min, then a 100 μL of N-(1-naphthyl) ethylenediamine was added to the mixture. The absorbance of the resultant solution was measured at 540 nm.

#### Acetylcholinesterase activity

The activity of acetylcholinesterase (AchE) was determined according to the method of Gorun et al.^32^. A 10 μL of tissue sample was added to a 50 μL of the enzyme’s substrate (acetylcholine) and a 140 μL of 20 mM phosphate buffer (pH 7.6). The mixture was incubated at 38 °C for 10 min. 1800 μL of 5,5′dithiobis (2-nitrobenzoic acid) was then added. The absorbance of the resultant solution was measured at 415 nm.

#### Measurements of monoamines neurotransmitters

The right half of the cortex was homogenized in 3 mL of an ice-cold solution of acidified n-butanol. The homogenates were centrifuged at 5000 rpm for 5 min. 2.5 mL of the supernatant was added to 1.6 mL of (0.2 N) acetic acid and 5 mL of heptane. The mixture was centrifuged again at 5000 rpm for 5 min to separate the aqueous layer from the alcoholic layer. The aqueous part was used for the estimation of dopamine (DA), norepinephrine (NE), and serotonin (5-hydroxytryptamine; 5H-T) according to the fluorometric method described by Ciarlone^33^. The fluorescence of DA, NE, and 5H-T was measured using a spectrofluorometer (model Jasco-FP-6500, Japan) with a source of xenon arc lamp 150 W at different excitation and emission wavelengths. DA was measured at 320 nm and 370 nm; NE was measured at 380 nm and 460 nm and 5H-T was measured at 355 and 470 nm.

### Statistical analysis

The obtained data were analyzed statistically by one-way ANOVA followed by Duncan’s post hoc to compare different groups. The difference between groups was considered significant at P-value < 0.05. Data are presented as the mean ± SEM. To perform these analyses SPSS v26.0 was used.

### Ethics approval

This study was performed in line with the principles of the Declaration of Helsinki and approval was granted by the local Ethics Committee of Cairo University under the number CU / IF/62/18. The study is reported in accordance with ARRIVE guidelines.

## Results

### Characterization of silver nanoparticles

#### UV–visible spectrophotometry

**Figure 2:**
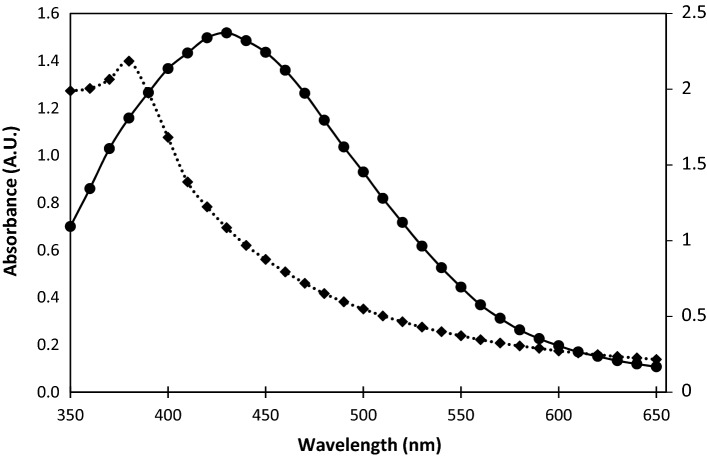
UV–Vis. spectrum of green synthesized AgNPs (solid line) showing a peak absorbance at 430 nm and of PGLE (dotted line) showing a peak at 380 nm.

The UV–Vis spectrum of the green synthesized AgNPs showed an absorbance peak at 430 nm (Fig. 2).

#### Fourier transformed infrared (FTIR) spectroscopy

**Figure 3:**
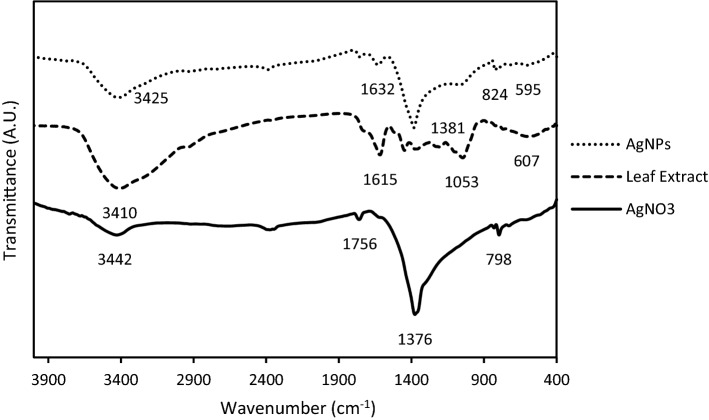
FTIR spectrum of the AgNO_3_ solution, PGLE, and the resultant biosynthesized AgNPs.

The FTIR spectrum (Fig. 3) of the green synthesized AgNPs, along with the spectrum of both the AgNO_3_ and the PGLE showed peaks of AgNO_3_ that appeared at 3430 cm^−1^ (O–H stretch), 1760 cm^−1^ (C=O), 1380 cm^−1^ (characteristic to silver metal), 800 cm^−1^ (alkene), while peaks of PGLE appeared at 3410 cm^−1^ (O–H stretch), 1620 cm^−1^ (C=C stretch), 1050 cm^−1^ (C–O), 605 cm^−1^ (Aromatic ring), and peaks of AgNPs appeared at 3420 cm^−1^ (O–H stretch), 1630 cm^−1^ (C=C stretch), 1380 cm^−1^ (characteristic to silver metal), 820 cm^−1^ (alkenes) and 600 cm^−1^ (Aromatic ring). These results show shifts of signature peaks of both solutions in the green synthesized AgNPs with decreased intensity of the peaks indicating accommodation of functional groups from both solutions and a cap of flavonoids.

#### Transmission electron microscopy (TEM)

**Figure 4:**
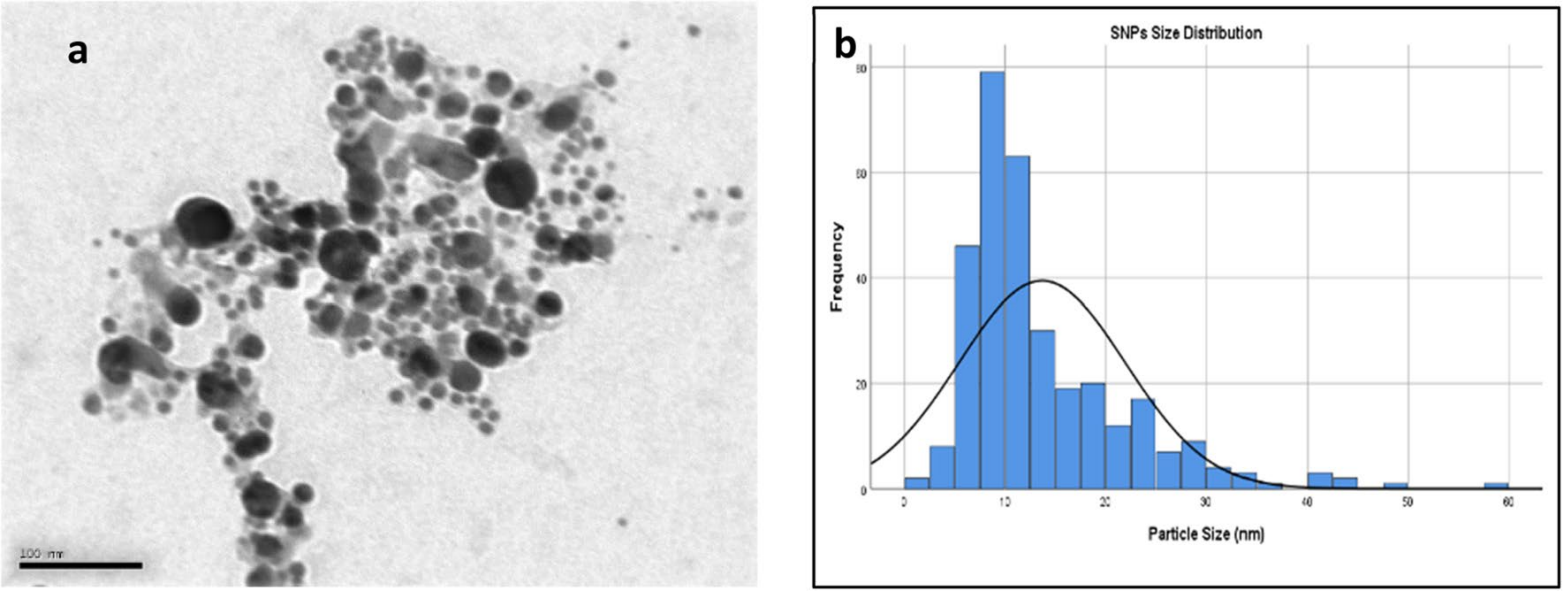
Average size and size distribution of the green synthesized particles detected by TEM image and IMAGEJ software.

The TEM image of the characterized AgNPs shows that the nanoparticles are spherical with an average diameter of 14 nm with a normal size distribution shown in Fig. 4.

#### Dynamic light scattering (DLS)

**Figure 5:**
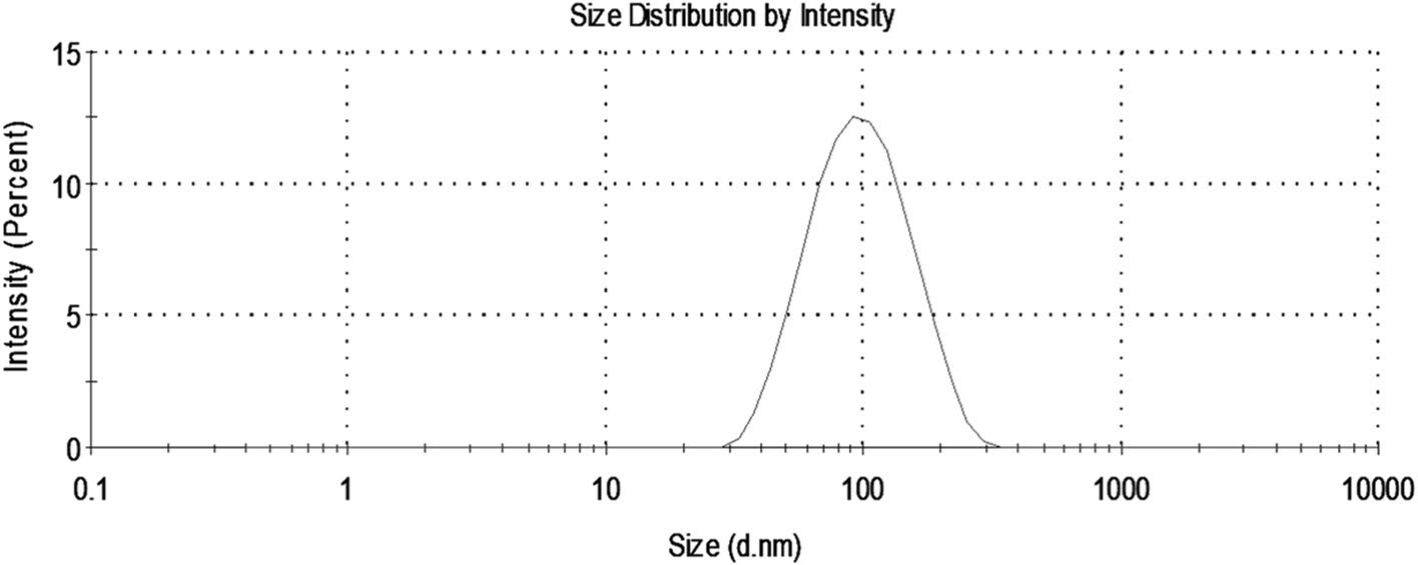
Size distribution of green synthesized AgNPs by DLS.

The size of the particles in a solution also known as its hydrodynamic size was measured by dynamic light scattering. The green synthesized AgNPs showed an average hydrodynamic size of 86.58 nm with a polydisperse index (PDI) of 0.178 (Fig. 5).

#### Zeta potential

**Figure 6:**
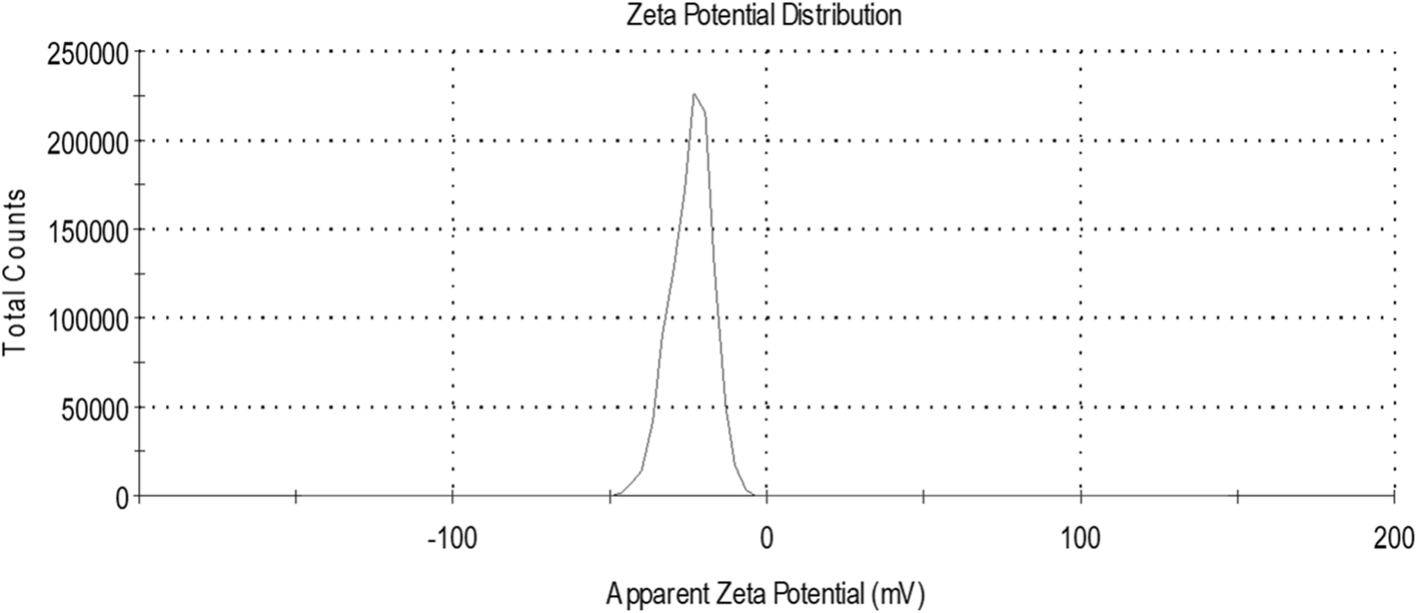
Zeta potential of the green synthesized AgNPs.

The measured zeta potential of the green synthesized AgNPs indicates their stability in the solution which therefore indicates their stability in the biofluids. The zeta potential detected for these nanoparticles was − 24 ± 6.52 mV (Fig. 6).

### Ultrastructural analysis of brain tissues

**Figure 7:**
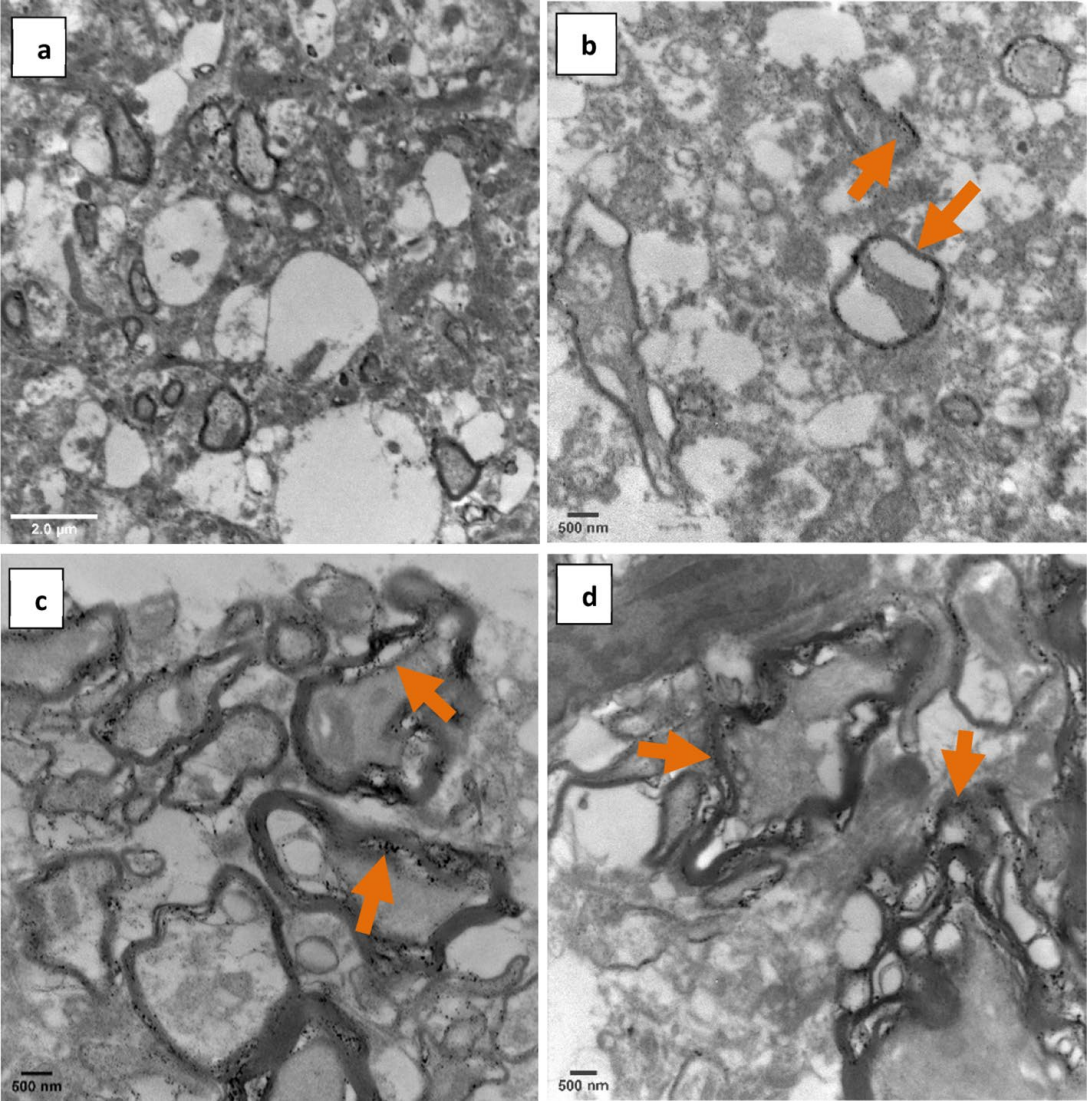
TEM Micrographs of AgNPs in brain tissues: (**a**) Control group, at magnification ×15,000 and scale bar 2.0 μm, shows no change in the ultrastructure of myelinated axons (white arrows). (**b**) The low dose group (0.5 mg/kg), at magnification ×12,000 and scale bar 500 nm, shows slight structural changes in the myelinated axons and their cytoplasm (orange arrows). (**c**,**d**): Medium dose group (5 mg/kg), at magnification ×12,000 and ×10,000 respectively, and scale bar 500 nm, shows higher concentrations of biosynthesized AgNPs in the myelin lamella and their increased structural disturbance (orange arrows).

The histological analysis of the brain tissues due to the administered AgNPs doses was detected by TEM. Figure 7a–d shows the micrographs of brain tissues from the different administered doses of the low (0.5 mg/kg) and medium (5 mg/kg) doses against the control group. These doses were chosen for TEM imaging, rather than the high dose (10 mg/kg), because they showed slight changes, in comparison to the high dose, in the biochemical parameters, therefore they were the focus of the TEM investigation for a closer screening of their structural effects. The micrographs, confirm the presence of the AgNPs in the brain tissues and their concentration in the myelin sheath of neurons. In addition, the low dose had a small ultrastructural change on the myelin membranes of the axons and their axoplasm (cytoplasm) (Fig. 7b), whereas the medium dose caused more observable structural changes to the neurons and their myelinated membranes causing a clear disruption to their structure (Fig. 7c,d) in comparison to the control group (Fig. 7a). On the other hand, the control group showed no such changes in the axoplasm or the myelin structure of the neurons.

### Oxidative stress

**Figure 8:**
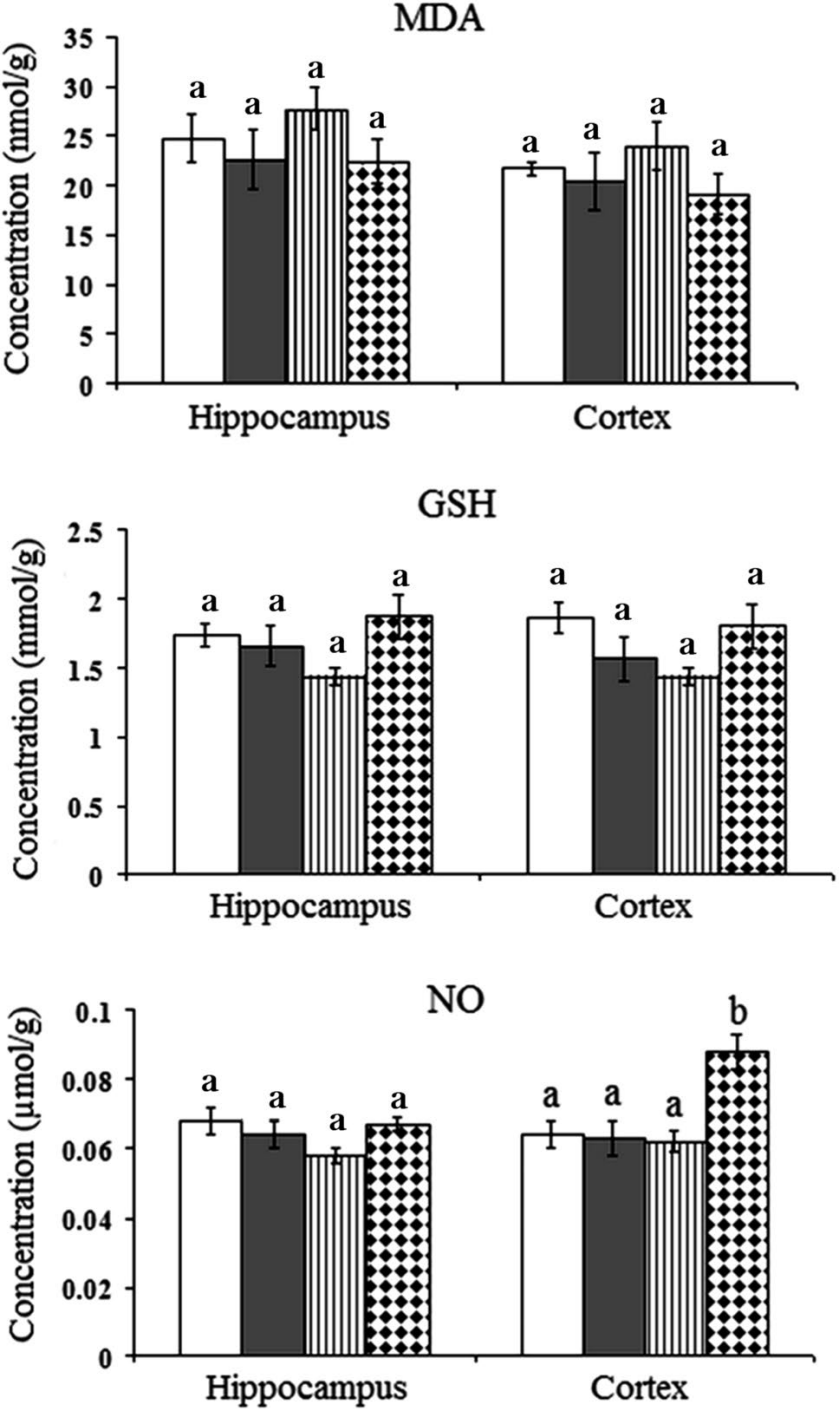
Effect of different doses of green synthesized silver nanoparticles (AgNPs) (0.5 mg/kg, 5 mg/kg, and 10 mg/kg) on the levels of lipid peroxidation (MDA), reduced glutathione (GSH), nitric oxide level (NO) in the hippocampus and cortex of rat brain.  Control,  Rats treated with AgNPs (0.5 mg/kg),  Rats treated with AgNPs (5 mg/kg),  Rats treated with AgNPs (10 mg/kg). Statistically significant means (p-value < 0.05) are given different letters and the same letters indicate non-significant changes.

ANOVA revealed no significant changes in lipid peroxidation levels between the control group and groups treated with AgNPs (P-values > 0.05). The cortex and hippocampus of rats treated with any of the three doses of AgNPs (0.5, 5, and 10 mg/kg) showed control-like values of MDA. Similarly, the cortical and hippocampal levels of reduced glutathione showed no significant difference between control animals and animals treated with AgNPs at different doses (P-values > 0.05). Regarding the NO results, ANOVA revealed a significant increase in the cortical level of NO when rats were treated with a high dose of AgNPs (10 mg/kg) when compared to the control value (P-value < 0.05). This increase recorded 38% of the control value. Meanwhile, the NO level exhibited a control-like value in the hippocampus of rats treated with 10 mg/kg of AgNPs. In the cortex and hippocampus, no significant changes were recorded in NO levels when rats were treated with 0.5 mg/kg and 5 mg/kg of AgNPs (Fig. 8).

### Acetylcholinesterase activity

**Figure 9:**
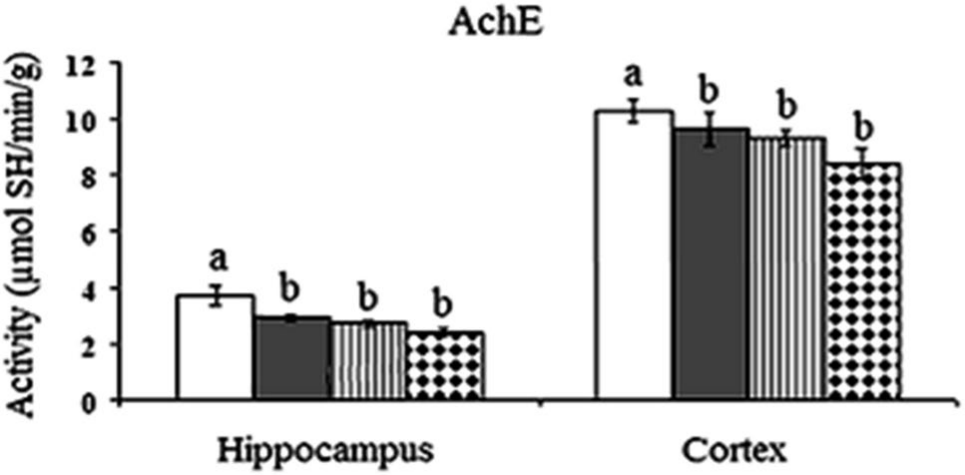
Effect of different doses of green synthesized silver nanoparticles (AgNPs) (0.5 mg/kg, 5 mg/kg, and 10 mg/kg) on the activity of acetylcholinesterase (AchE) hippocampus and cortex of rat brain.  Control,  Rats treated with AgNPs (0.5 mg/kg),  Rats treated with AgNPs (5 mg/kg),  Rats treated with AgNPs (10 mg/kg). Statistically significant means (p-value < 0.05) are given different letters and the same letters indicate non-significant changes.

ANOVA revealed significant changes in the cortical and hippocampal AchE activity between control animals and animals treated daily for 14 days with the three doses of AgNPs (P-value < 0.05). In the cortex of rats treated with 0.5 mg/kg, 5 mg/kg and 10 mg/kg, AchE activity decreased significantly recording 6%, 9%, and 18%, respectively lower than control rats. In the hippocampus, AchE activity showed a significant decrease in rats treated with any of the three doses of AgNPs recording 21%, 26%, and 35%, respectively lower than the control value (Fig. 9).

### Monoamines levels

**Figure 10:**
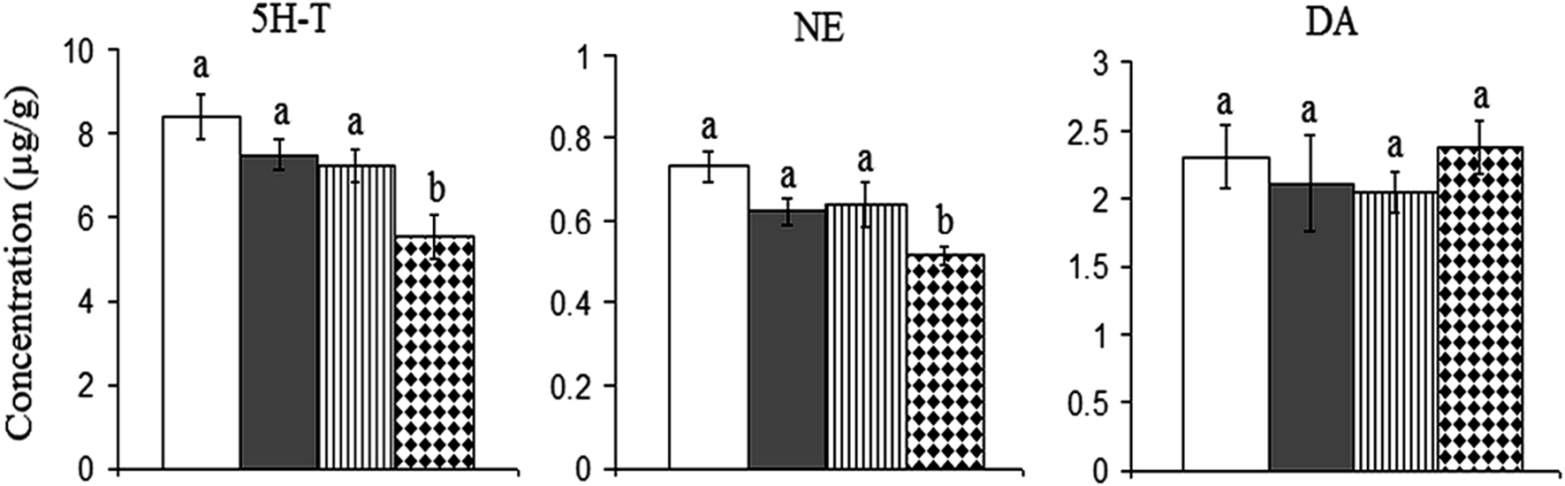
Effect of different doses of green synthesized silver nanoparticles (AgNPs) (0.5 mg/kg, 5 mg/kg, and 10 mg/kg) on the levels of serotonin (5H-T), norepinephrine (NE), and dopamine (DA) in the cortex of rat brain.  Control,  Rats treated with AgNPs (0.5 mg/kg),  Rats treated with AgNPs (5 mg/kg),  Rats treated with AgNPs (10 mg/kg). Statistically significant means (p-value < 0.05) are given different letters and the same letters indicate non-significant changes.

The cortical levels of 5H-T, NE, and DA showed non-significant changes when rats were daily administered with 0.5 mg/kg or 5 mg/kg of AgNPs for 14 days. However, the daily treatment for the same period with 10 mg/kg of AgNPs resulted in a significant decrease in the cortical levels of 5H-T and NE recording 34% and 30%, respectively, lower than control values. However, the cortical dopamine showed a control-like value with the later dose of AgNPs (Fig. 10).

## Discussion

Due to the wide use of AgNPs in several fields such as in biomedical applications^34^, consumer products packaging^35^, and the water purification process^36^, several trials have been made to minimize the toxicity of AgNPs and boost their benefits to biological systems. One strategy to minimize toxicity is carried out by green or biosynthesis of AgNPs. However, the possible toxicities of these nanoparticles on biological tissues have not yet been fully investigated. Thus, the current study was designed and conducted to evaluate the neurochemical and structural changes induced by the green synthesized AgNPs from *P. guajava* leaves on rat’s brain.

Several studies have reported neurotoxicity of AgNPs that include oxidative stress, neuroinflammation, and neurodegeneration^12–14^. It has been reported that biological methods are preferred for being cost-effective and eco-friendly, as they don’t involve the use of toxic chemicals. Furthermore, nanoparticle green synthesis is not time-consuming compared to other biological processes^37^. This strategy has attracted more attention among the most recent advances, particularly for silver (Ag) and gold (Au) NPs synthesis, which is more secure in comparison to other metallic NPs^38^. Synthesis of AgNPs using different medicinal plants for pharmaceutical and biological applications has been reported by Li et al.^39^, Leela and Vivekanandan^40^, Song and Kim^41^, and Mani et al.^42^.

It has been observed that plant extract can be used for the green synthesis of metallic nanoparticles depending on the reducing properties of this extract^43^. In the present study, AgNPs were prepared using PGLE. This method depends on the presence of polar secondary metabolites including glycosides and polyphenolics like flavonoids and tannins in the extract that could be responsible for the reduction of metal ions to nanoparticles^44^. Previous studies have reported that PGLE produces silver nanoparticles in the range of 1–100 nm^18–21^, which is largely affected by the synthesis parameters. Therefore, different parameters were used in the current study to control and optimize the size of the green synthesized AgNPs to the size that also fulfills an optimum concentration of the doses. These parameters were: AgNO_3_ molarity, pH, temperature, and PGLE volume added^45^. The parameters were optimized through different synthesis trials and the optimum molarity of AgNO_3_ solution, pH, temperature, and PGLE volume that produced the administered AgNPs were 5 mM, 7, 90 °C, and 0.2 mL, respectively. These values were favored according to the stability and the produced size of the AgNPs.

Characterization of the biosynthesized AgNPs is a major marker of their stability, biodispersity, agglomeration, and biofunctionalization in the tissues. Therefore, nanoparticles were characterized by UV–Vis Spectroscopy, FTIR, DLS, Zeta potential, and TEM. The TEM images showed that the prepared nanoparticles were spherical with an average size of 14 nm which was less than the size recorded by the DLS (86.58 nm). This difference in the recorded size between the two techniques could be attributed to surrounding leaf extract moieties and water particles attached to the nanoparticles in the case of DLS measurements^46,47^, in addition to the small aggregation of particles shown in the TEM image. Moreover, the DLS recorded a polydispersity index (PDI) of 0.178 which indicates the homogeneity and good dispersion of the biosynthesized AgNPs in the solution since PDI ranges from a value of 0.01 to 0.5 for monodispersed particles. Samples with a very broad size distribution have PDI value > 0.7^48^. PDI value in the range of 0.1 – 0.4 indicates that the preparation has a moderate disperse distribution^49^.

Metal nanoparticles have free electrons, which yield a surface plasmon resonance absorption band, due to the mutual vibration of electrons of metal nanoparticles in resonance with the light wave^39^. The plasmon resonance peak (PRP) detected by the UV–Vis spectrophotometry appeared at 430 nm, which is in line with the results of Le et al.^16^, supporting the reduction and presence of AgNPs in the solution.

FTIR has become an important tool in understanding the involvement of functional groups in the relation between metal particles and biomolecules. FTIR spectral measurements were carried out to identify the potential biomolecules in PGLE which is responsible for reducing, capping, and stabilizing the reduced silver nanoparticles^16^. FTIR showed the structure, the respective bands of the synthesized nanoparticles, and the stretch of bonds. Therefore, the FTIR result of the AgNO_3_ solution, PGLE, and the AgNPs show that AgNPs have accommodated peaks from both, the AgNO_3_ and the PGLE solutions with slight shifting in the position and moderation in the intensity. This indicates the synthesis of the AgNPs carrying part of the PGLE features (functional groups), mainly as a coating. This is clear from the several shifts of the signature peaks of both solutions spectra that appeared in the AgNPs spectrum. One of these accommodated (shifted) peaks are the peaks of flavonoids and tannins in the PGLE and AgNPs proving the gain of flavonoids and tannins as capping and stabilizing agents during the synthesis process over and above as a reducing agent as it is proved to be the primary reducing agent in the PGLE contents^5,50^. The intensity and position of the AgNPs decreased and shifted, respectively, mainly due to the consumption of the groups during the biosynthesis reduction process, this finding is in agreement with the results of Ali et al.^51^.

The zeta potential (ZP) values of the AgNPs were − 24 ± 6.52 mV. The relatively high negative charges prevent the high agglomeration of the nanoparticles and keep the stability of the AgNPs synthesized in the present study. It has been demonstrated that particles tend to aggregate in the ZP range 0–5 mV; in the range 5–20 mV, particles are minimally stable; and particles are stable in the range 20–40 mV and highly stable in the range more than 40 mV^52^. This proves that the obtained AgNPs are stable and could stand for almost a month^51^. Moreover, the negative charge strongly refers to the capping agents present on the biosynthesized AgNPs^53,54^. In addition, the negative charges inhibit the tendency of these nanoparticles to interact with the negatively charged plasma protein. This could improve the pharmacokinetic properties of AgNPs by increasing the time of circulation. On the other hand, the biosynthesized AgNPs are expected to acquire an unavoidable protein corona in vivo, due to their exposure to plasma proteins in its route through the bloodstream, which would add further stabilization to the nanoparticles in the biological medium in addition to altering their biological activity^55,56^.

One of the main targets of synthesizing nanoparticles is to enable them to cross the different barriers including the blood–brain barrier (BBB) to be highly distributed throughout the body. In addition, the nanoparticles could be used in small doses to avoid the toxicity that may be exerted by the high doses of the native substances. It is clear from the TEM images of the brain that AgNPs were observed in the cerebral tissues indicating their ability to cross the BBB^25^. The images indicate the presence of the AgNPs mostly in the myelin lamellar layers and other membranes of the astrocytes, cytoplasm, and blood vessels in the brain tissue, causing deformations in their ultrastructure. The present findings were in line with Skalska et al.^23^, Mohamed et al.^57^, and Opris et al.^58^ who observed the presence of AgNPs in lamellar layers of the myelin sheath of axons and blood vessel walls, causing ultrastructural changes in such cells and tissues. These findings also may give a further glimpse on the mode of AgNPs entry through the BBB. According to a review by Skalska and Struzynska^7^, AgNPs cause a disruption in the tight junctions of the BBB, which increases its permeability. This was shown and proven by the other membranes of the cells beyond the BBB in the TEM images. Moreover, the examination of cerebral tissues showed also that the abundance of the biosynthesized AgNPs was linked with dose. The lower dose shows less AgNPs abundance than the higher dose. This was further confirmed by Opris et al.^58^ who also added that these changes were not only dose-dependent but time-dependent too. The present distribution of green synthesized AgNPs inside the brain could be attributed to the affinity of the flavonoids cap and silver to sulfur and phosphorus elements^59,60^, which are present mostly in different proteins forming these membranes (walls) and the myelin sheath layers in addition to the proteins that act as enzymes or have other functions in the cytoplasm^56^.

Although several studies have reported that the neurotoxicity induced by AgNPs are mediated by oxidative stress, the present data showed no signs of oxidative stress in the hippocampus and cortex of rats treated with three different doses of the green synthesized AgNPs. This was evident from the normal-like levels of lipid peroxidation, and reduced glutathione and nitric oxide in the studied brain regions. The cerebral tissues are highly sensitive to oxidative stress due to the high concentration of polyunsaturated fatty acids that exist in membrane lipids in the brain^61^. Polyunsaturated fatty acids especially serve as major biological targets for oxidative damage induced by ROS. This in turn increases the level of malondialdehyde, one of the final products of polyunsaturated fatty acids peroxidation in the cells which represents the marker of lipid peroxidation^62^. In addition, reduced glutathione (GSH) acts as the primary intracellular antioxidant and redox buffer^63^. Therefore, the reduced level of GSH could be due to its use as a free radical scavenger. The unchanged levels of these two parameters indicate the safety of the present green synthesized AgNPs formula on the oxidative status of the tissue.

Nitric oxide levels, however, increased only in the cortex when rats were treated with the high dose of AgNPs (10 mg/kg), whereas no significant changes were observed with the other two doses (0.5 mg/kg and 5 mg/kg). In the hippocampus NO showed minimal changes with the three applied doses of AgNPs. This increased NO level in the cortex though, cannot be interpreted as an oxidative stress marker since the other markers of oxidative stress did not show any change. Hence, this increase in the NO level can be attributed to the other functions of the NO in the central nervous system such as a neurotransmitter and its involvement in the process of memory, since the cortex and hippocampus are involved in the memory functions^64,65^. Alternatively, the role of NO as a vasodilator^66^ may increase cerebral blood flow. These findings prove the absence of oxidative stress in the cerebral tissues of rats treated with green synthesized AgNPs. These data are in agreement with the findings of Alkhalaf et al.^67^. The absence of oxidative stress can be interpreted by two mechanisms. Firstly, the capping of AgNPs with flavonoids gained from the PGLE protects the silver surface from oxidation reaction by sequestering AgNPs surfaces and their released Ag^+^^68^. Secondly, green synthesized AgNPs are capped with flavonoids, including quercetins, that have potent antioxidant characteristics and their ability to ameliorate oxidative damage^17,18,50,59,69^. These two interpretations are further supported by Suthar et al.^56^, stating that NP coating is used to improve stability by reducing agglomeration, minimizing oxidation, and restricting ionic release. And that Several studies have discovered a relationship between coating characteristics and toxicity.

The present data showed that the three doses of the green synthesized AgNPs (0.5, 5, and 10 mg/kg) decreased the activity of AchE, the metabolizing enzyme of acetylcholine (Ach), in the cortex and hippocampus. This decrease was more obvious in the hippocampus than the cortex, especially with low and medium doses. The decreased AchE activity might be due to the effect of the flavonoid cap of the green synthesized AgNPs in addition to the nanoparticles themselves, since flavonoids are non-competitive inhibitors of the AchE enzyme especially quercetin^70,71^, which is proved to be present in the PGLE^5^. The present results are in agreement with the study of Youssif et al.^72^ who found a decreased AchE activity in rats treated with green synthesized AgNPs and Vanin dos Santos Lima et al.^73^ who also found an inhibition effect of green synthesized AgNPs on AchE in vitro. Moreover, the results of Marinho et al.^74^ further support this inhibition effect; since he reported such an inhibition effect by chemically synthesized AgNPs on AchE activity in the brain and muscles of zebrafish. Acetylcholine is one of the important neurotransmitters in the brain that plays a major role in memory and cognitive function. The reduced level of Ach has been reported to mediate dementia and memory impairment in Alzheimer’s patients^75^.

The present treatment of AgNPs at the doses of 0.5 and 5 mg/kg showed non-significant changes in the cortical monoamine neurotransmitters (5H-T, NE, and DA) levels with respect to control values. However, the high dose (10 mg/kg) reduced the 5H-T and NE significantly. It is well known that the changes in monoamine neurotransmitters have a potential role in many psychiatric diseases and mood disorders. The decreased levels of 5H-T and/or NE are involved in the pathogenesis of depression^76^. The decrease in DA is the main reason for Parkinson’s disease^77^. Therefore, the unchanged levels of monoamines observed with the low and moderate doses of AgNPs support the evidence of the safety of the green synthesized AgNPs. However, the decreased 5H-T and NE recorded with the high dose could mediate several pathological conditions. It has been reported that AgNPs had a stimulatory effect on MAO-A mRNA expression in pups only at the age of 7 and 14^78^. Although the study of Tabatabaie et al.^78^ investigated the toxicity of AgNPs on monoamine oxidase-A which is responsible for the breakdown of catecholamines^79^, their toxicity on monoamine oxidase-B, the enzyme responsible for the breakdown of serotonin can’t be excluded. Therefore, the present decrease in serotonin and norepinephrine due to the high dose of AgNPs could be attributed to the increased MAO activity. Thus, the present high dose of AgNPs (10 mg/kg) may be the onset dose at which the toxicity of AgNPs occur.

## Conclusions

The present findings showed that the green synthesis of AgNPs gained several advantageous physicochemical characteristics that reflected on its biological impacts. Firstly, it reduces the silver nitrated toxicity to a great extent. In addition, the capping of the nanoparticles with flavonoids and quercetin prevents the oxidative stress that may be induced by silver. Moreover, the ability of the green synthesized AgNPs to cross the blood–brain barriers may allow the use of these particles, with such safer features, as a brain drug delivering system. However, the inhibitory action that these green synthesized nanoparticles exerted on the cortical and hippocampal AchE activity and the significant attenuation of 5H-T and NE at a 10 mg/kg dose of AgNPs may render these nanoparticles toxic and a precautionary approach should be followed.

## Data Availability

The datasets generated during and/or analyzed during the current study are available from the corresponding author on reasonable request.
